# Desired dementia care towards end of life: Development and experiences of implementing a new approach to improve person‐centred dementia care

**DOI:** 10.1111/jan.16285

**Published:** 2024-06-24

**Authors:** Jesper M. A. Biesmans, Sascha R. Bolt, Daisy J. A. Janssen, Toon Wintjens, Chandni Khemai, Jos M. G. A. Schols, Jenny T. Van Der Steen, Sandra M. G. Zwakhalen, Judith M. M. Meijers

**Affiliations:** ^1^ Department of Health Services Research Maastricht University, Care and Public Health Research Institute Maastricht The Netherlands; ^2^ Living Lab for Aging and Long‐Term Care Limburg, Department of Health Services Research Maastricht University Maastricht The Netherlands; ^3^ Zuyderland Medical Center Sittard‐Geleen The Netherlands; ^4^ Tranzo, Tilburg School of Social and Behavioral Sciences Tilburg University Tilburg The Netherlands; ^5^ Department of Family Medicine, Care and Public Health Research Institute, Faculty of Health, Medicine and Life Sciences Maastricht University Maastricht The Netherlands; ^6^ Department of Public Health and Primary Care Leiden University Medical Center Leiden The Netherlands; ^7^ Department of Primary and Community Care Radboud University Medical Center Nijmegen The Netherlands; ^8^ Radboudumc Alzheimer Center Radboud University Medical Center Nijmegen The Netherlands

**Keywords:** dementia, nurse education, nursing home care, palliative care, research implementation

## Abstract

**Aims:**

To describe the co‐creation of the ‘Desired Dementia Care Towards End of Life’ (DEDICATED) approach to improve person‐centred palliative care for individuals with dementia and to describe the experiences of healthcare professionals during the approach's implementation.

**Methods:**

A needs assessment, comprising both qualitative and quantitative studies, informed palliative care needs of healthcare professionals, family caregivers and individuals with dementia. The approach was co‐created with healthcare and education professionals, guided by the findings. Then, healthcare professionals were trained to implement the approach in their organizations. From April to June 2022, semi‐structured interviews with actively engaged professionals were analysed using Conventional Content Analysis.

**Results:**

The needs assessment yielded six key themes: (1) raising palliative care awareness, (2) familiarization with a person with dementia, (3) communication about future care preferences, (4) managing pain and responsive behaviour, (5) enhancing interprofessional collaboration in advance care planning and (6) improving interprofessional collaboration during transitions to nursing homes. Interviews with 17 healthcare professionals revealed that active involvement in co‐creating or providing feedback facilitated implementation. Overall, the DEDICATED approach was perceived as a valuable toolkit for optimizing palliative care for people with dementia and their loved ones.

**Conclusion:**

Co‐creating the DEDICATED approach with healthcare professionals facilitated implementation in daily practice. The approach was considered helpful in enhancing person‐centred palliative dementia care.

**Impact Statement:**

This study underscores the importance of active involvement of healthcare professionals in the research and development of new interventions or tools for palliative care, which can influence the successful implementation, dissemination and sustained usage of the developed tools.

**Implications for the Profession and Patient Care:**

The developed approach can improve person‐centred palliative care for individuals with dementia, ultimately improving their quality of life and that of their loved ones.

**Reporting Method:**

This study used the Consolidated Criteria for Reporting Qualitative Research.

**Patient of Public Contribution:**

No patient or public contribution.

## 1 INTRODUCTION

Dementia is an incurable chronic condition that places a substantial burden on both individuals afflicted and their families (Chiao et al., 2015; Ibrahim et al., 2017). The global population living with dementia is projected to reach approximately 150 million by 2050 (Prince et al., 2015). Palliative care aims to improve the quality of life for individuals with life‐limiting conditions such as dementia, by centralizing the wishes, needs and values of both the individual with dementia and their loved ones (Brinkman‐Stoppelenburg et al., 2014; Buss et al., 2017). As dementia leads to progressive cognitive decline, conducting timely advance care planning (ACP) conversations is an important aspect of palliative care. ACP is the process of communication between the healthcare professionals (HCPs), the person with dementia and their loved ones about personal values, goals and preferences regarding future care and treatment. Ideally, ACP conversations are initiated early after the diagnosis of dementia, repeated as the disease progresses according to needs and continued with loved ones when the person with dementia becomes unable to participate in decision‐making (Eisenmann et al., 2020; Rietjens et al., 2017; Smeenk et al., 2017; van der Steen et al., 2023).

## 2 BACKGROUND

Many challenges remain in providing high‐quality palliative care for people with dementia. For example, the lack of readiness of people with dementia and their loved ones to discuss their wishes, needs and preferences can be a hurdle for HCPs to initiate ACP conversations (Dening, 2018; Zwakman et al., 2021). Additionally, discussing these wishes, needs and preferences for palliative care can be confronting and emotional (Bloomer et al., 2010). Moreover, HCPs may not feel ready or confident to start these conversations, or they might lack sufficient knowledge or literacy about what palliative dementia care entails (Coffey et al., 2014; Dening, 2018; Peerboom et al., 2023; Smith et al., 2023). Lastly, interprofessional collaboration when providing palliative care is often perceived as challenging by HCPs (Dening et al., 2018). Many healthcare disciplines are involved in the care process leading to difficulties in communication and coordination between HCPs and with loved ones (Khemai et al., 2022). These challenges highlight the necessity of developing needs‐based education and support for HCPs in the field of palliative dementia care (Midtbust et al., 2018; Schroeder & Lorenz, 2018). This might help to improve the quality of palliative care provided to people with dementia.

While evidence‐based tools are available to support HCPs when providing palliative care, some do not align well with the practical context of palliative care provision, which hinders the effective use of these interventions (van Riet Paap et al., 2015). Therefore, there is increasing recognition of the benefits of using co‐creative methods, as involving end‐users in the development of tailored tools can lead to improved value, impact and implementation of an intervention (Frow et al., 2016; Jull et al., 2017).

The DEDICATED project (Desired Dementia Care Towards End of Life) aims to provide HCPs (such as nurses and dementia case managers) with practical tools and guidance for the provision of palliative dementia care. The complete set of tools and guidance is called the DEDICATED approach and was developed in co‐creation with HCPs and educators in the field of palliative care for people with dementia. The key objectives of the DEDICATED approach include (1) raising awareness among HCPs about the palliative care needs of people with dementia; (2) facilitating communication between HCPs and people with dementia and their loved ones about wishes, needs and values as part of ACP; (3) providing HCPs with guidance in managing pain and responsive behaviour and (4) fostering collaboration between the person with dementia, their loved ones and HCPs, and across care settings. To disseminate and implement the DEDICATED approach, DEDICATED ambassadors were trained. DEDICATED ambassadors are HCPs who play an important role in stimulating awareness about palliative care and dementia, introducing the DEDICATED approach to their colleagues and sustaining the application of the DEDICATED approach in practice.

The objective of this study is twofold. First, to provide a detailed description of the development of the DEDICATED approach. Second, to describe the experiences of co‐creating, using and implementing the DEDICATED approach in daily practice.

## 3 THE STUDY

### 3.1 Aims

**FIGURE 1:**
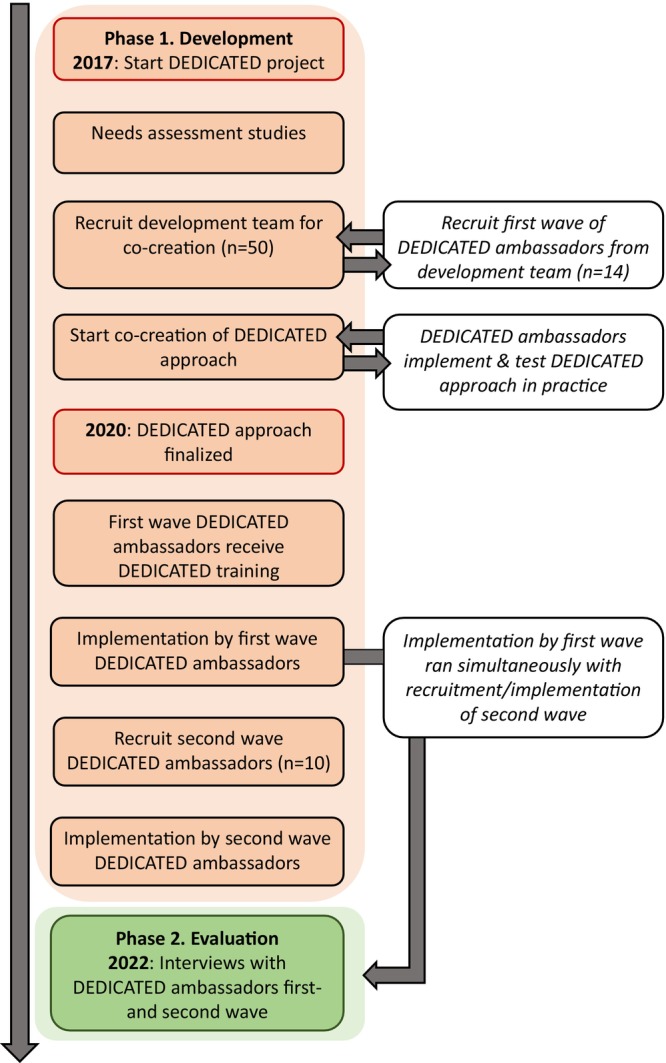
Complete timeline for the development and evaluation of the DEDICATED approach.

This study describes how the DEDICATED approach was developed and evaluated using a co‐creation approach. Co‐creation involves stakeholders at all stages of an initiative, from the problem identification and solution generation through to implementation and evaluation (Vargas et al., 2022). This paper is structured into two sections, which together describe the complete co‐creation of the DEDICATED approach. Part 1 describes how the DEDICATED approach was developed using iterative development cycles. Part 2 describes the evaluation of the experiences of HCPs (DEDICATED ambassadors) who implemented this developed DEDICATED approach in their daily practice. Figure 1 depicts the complete timeline of the development and implementation process of the DEDICATED approach.

### 3.2 Part 1: Development of the DEDICATED approach

The development step of the DEDICATED approach consists of two sub‐steps: (1) a needs assessment and (2) co‐creation of the DEDICATED approach.

#### 3.2.1 Needs assessment

The first step, a broad assessment of palliative care needs, involved a series of qualitative and quantitative studies involving (1) people with dementia, (2) family caregivers and (3) HCPs. The results of the needs assessment have been published elsewhere (Bolt et al., 2020, 2022; Bolt, van der Steen, Schols, Zwakhalen, & Meijers, 2019; Bolt, van der Steen, Schols, Zwakhalen, Pieters et al., 2019; Bolt, Verbeek et al., 2019; Khemai et al., 2020, 2022). After completion of the needs assessment studies, two researchers (SB and CK) conducted a thematic analysis of the results of these studies. The results were discussed in two meetings with six research team members, resulting in six overall themes. Then, these themes were discussed within two DEDICATED working groups, which consisted of patient representatives, family caregivers, researchers, HCPs and lecturers (*n* = 21). This led to a further refinement of the wording of the themes. Afterwards, an advisory board discussed the refined themes. The advisory board consisted of members of the Dutch Alzheimer Foundation, the Netherlands Comprehensive Cancer Organization, the Dutch Nurses Association and directors of from healthcare and educational organizations which were part of the Living Lab of Ageing and Long‐term Care Limburg (*n* = 24). Lastly, the refined themes were discussed with the DEDICATED development team. The development team consisted of HCPs (such as nurses and nursing lecturers) and were directly involved in co‐creating the DEDICATED approach. Eventually, this led to the following six overall themes:

1. awareness about the need for palliative care in dementia,
2. familiarization with a person with dementia/with each other (e.g. get to know the biography, values and behaviour of the person with dementia and loved ones to provide person‐centred palliative care),
3. communication about (future) care preferences as a part of ACP,
4. interprofessional collaboration in ACP,
5. interprofessional collaboration during care transitions,
6. managing pain and responsive behaviour (e.g. how HCPs can respond when a person with dementia is (potentially) in pain or behaves in a way that is experienced as challenging).

#### 3.2.2 Co‐creating the DEDICATED approach

**FIGURE 2:**
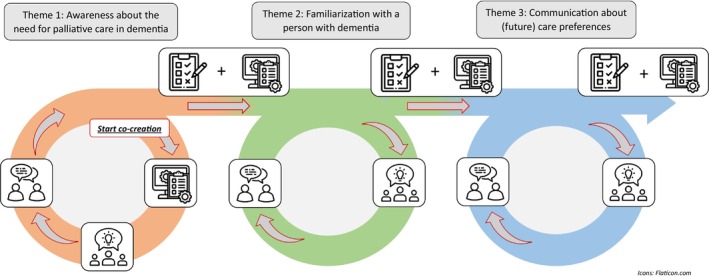
Three iterative development cycles with co‐creation sessions and testing/implementation.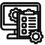 = Inventory existing tools: The development team inventoried existing support tools and methods related to the key themes and brainstormed potential modifications to improve the suitability of the tools for practice.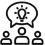 = Brainstorm for new/modified tools: The development team brainstormed about required support tools. The inventory and development process considered the organizational context by exploring each organization's current practices, possibilities and barriers concerning the use of existing and newly developed tools and methods.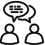 = Discussing developed tools: The developed tools were discussed and the development team provided feedback for refinement. After this session, the developed tools were tested in daily practice and evaluated within team members' own care wards or organizations.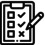 = Evaluate usage: DEDICATED–ambassadors pilot tested the tools in practice. After testing, the tools were evaluated by the development team to further refine or adapt the tools.

The DEDICATED approach was developed based on the aforementioned six themes using principles of co‐creation. Co‐creation is rooted in participatory action research (PAR), in which end‐users of a product or service are actively involved in the problem identification, development process and evaluation of an intervention (Bergdahl et al., 2019; Langley et al., 2018; Vargas et al., 2022). An underlying framework of PAR describes several important principles for co‐creation. For example, participants have a collective purpose to identify a problem/challenge and collaborate to find useful solutions for the identified problem. Importantly, participants (such as researchers and end‐users) build alliances to plan the dissemination and implementation of a co‐created intervention (Robert, 2013). The development team that participated in the needs assessment was directly involved in co‐creating and testing the DEDICATED approach in practice using iterative co‐creation cycles: for each of the six central themes, a co‐creation cycle was conducted. Figure 2 visualizes the actions taken by the development team per co‐creation cycle. In addition, the advisory board provided additional feedback on the developed tools (*n* = 21).

To test the developed tools in practice, HCPs (such as nurses) from the development team were invited to become a DEDICATED ambassador. DEDICATED ambassadors are HCPs who implement and promote the usage of the DEDICATED approach within their care organization. HCPs were eligible to become DEDICATED ambassadors if they were:

1. Working in one of the partner healthcare organizations of DEDICATED (e.g. a home care team or nursing home unit which was part of the Living Lab of Ageing and Long‐term Care Limburg);
2. Involved in palliative care provision for people with dementia.

### 3.3 Part 2: Methods for analysing the experiences with the DEDICATED approach

To evaluate the usage and implementation of the co‐created DEDICATED approach, we conducted individual online interviews with DEDICATED ambassadors from the first *and* second wave. This part of the study involved a descriptive qualitative methodology, as we attempted to describe the experiences of DEDICATED ambassadors when co‐creating and implementing the DEDICATED approach (Sandelowski, 2000). The DEDICATED ambassadors were invited to participate in these semi‐structured interviews from April to June 2022. We used consolidated criteria for reporting qualitative studies (COREQ) to describe the methods and findings of this part of the study (Tong et al., 2007).

#### 3.3.1 Population and recruitment

The evaluated population comprised DEDICATED ambassadors. We specifically chose to interview the DEDICATED ambassadors because they implemented the DEDICATED approach within their healthcare organizations/units. DEDICATED ambassadors were eligible to participate if:

1. they had participated in DEDICATED training sessions to become a DEDICATED ambassador and
2. they had direct contact with persons with dementia in their care practice.

The complete group of DEDICATED ambassadors (*n* = 24) was contacted by e‐mail to invite them to participate. After agreeing to participate, the DEDICATED ambassadors could then indicate their availability for the online interviews.

#### 3.3.2 Data collection

The semi‐structured interviews were held online and performed by members of the research group (JB, JM, SB, LD, CK and EK). An interview guide was used containing questions about how the DEDICATED ambassadors had experienced the training, tools and implementation of the DEDICATED approach. Supplement 1 shows the interview guide that was used during this study.

#### 3.3.3 Data analysis

The interview recordings were transcribed verbatim. To analyse the interview transcripts, we conducted a conventional content analysis (CCA). We let the open codes flow directly from our data, without any presumption formed before starting data analysis (Braun & Clarke, 2012; Hsieh & Shannon, 2005). A CCA involves open and axial coding (Braun & Clarke, 2012; Hsieh & Shannon, 2005). Open coding identified text fragments pertinent to the research question, while axial coding organized open codes into overarching categories (Hsieh & Shannon, 2005). The coding process was conducted using Atlas.ti software.

#### 3.3.4 Rigor

Two researchers individually coded the first two transcripts. The two researchers (JB and TW) then engaged in a reasoning session to discuss the coding process and exchange their findings to align the codes to the transcript data. Afterwards, the two researchers coded the remaining interviews individually using six reflection sessions to triangulate the coding process.

#### 3.3.5 Ethical considerations

The Medical Ethical Committee of Zuyderland Medical Centre (METCZ20180026, METCZ20190095) determined on the 29 July 2019 that this study was not subject to the Medical Research Involving Human Subjects Act. All interview participants gave verbal and written informed consent for participating. Data have been stored on secure servers of Maastricht University.

## 4 RESULTS

The results section comprises two parts. The first describes the DEDICATED approach development results, and the second describes the interview findings.

### 4.1 DEDICATED approach development results

The DEDICATED approach was co‐created with the development team and entails (1) the DEDICATED training to educate new DEDICATED ambassadors on implementing the DEDICATED approach and (2) tools, guidelines and information (in Dutch), linked to the six themes identified in the needs assessment.

#### 4.1.1 DEDICATED tools

**FIGURE 3:**
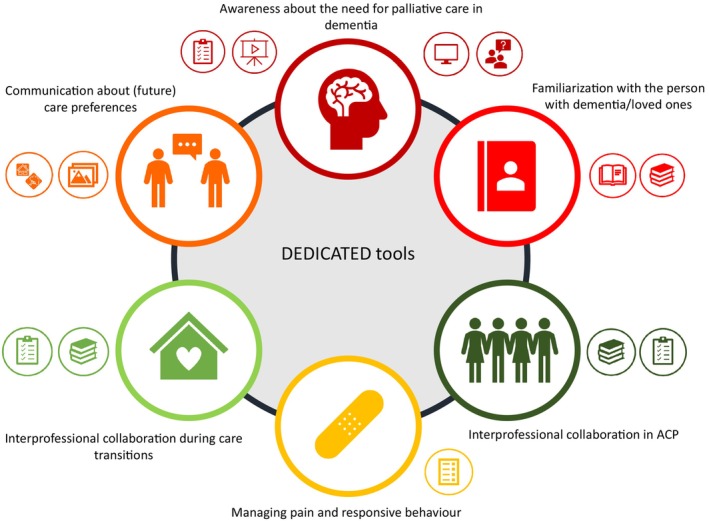
Visualization of the six themes and developed tools for each theme.

For each of the six central themes of the needs assessment, several physical and digital tools have been developed that can support HCPs during the provision of palliative care. For example, these tools can be used to initiate ACP conversations, guide HCPs during interprofessional collaboration or help HCPs identify pain and responsive behaviour. Figure 3 and Table 1 provide a detailed description of each tool.

**TABLE 1:** Overview of the developed DEDICATED approach.

Theme		Tools
Awareness about the need for palliative care in dementia	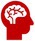	Short movie about the importance of person‐centred care for persons with dementia Movie about the importance of ACP and (interprofessional) collaboration Format for discussing a case or situation regarding palliative care for persons with dementia ‘Chatter jar’ with small cards containing reflective questions that can be used by a team to reflect on the provision of palliative care Reflection structure to reflect on situations and cases with a care team DEDICATED website providing information about palliative dementia care
Familiarization with a person with dementia/loved ones	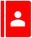	Book and poster describing the life of a person with dementia with the use of pictures and stories, assembled by their loved ones and HCPs Tools and guidelines that provide structure and guidance for loved ones of the person with dementia and HCPs to conduct ACP conversations and identify palliative care‐related wishes, needs and values
Communication about (future) care preferences as part of ACP	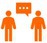	Photo cards that can be discussed with the person with dementia or involved loved ones. The information can be used for providing personalized care Dice with symbols that represent specific themes. The dice can be used to start conversations with people with dementia and/or their loved ones
Managing pain and responsive behaviour	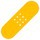	Revised version of the PACSLAC‐D to identify pain and responsive behaviour (including a short movie)
Interprofessional collaboration in ACP	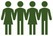	Conversation tools and guidelines that provide structure and guidance for loved ones of the person with dementia and HCPs to conduct ACP and identify wishes and preferences for palliative care
Interprofessional collaboration during care transitions	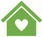	Guideline and infographic for interprofessional collaboration during the process of moving a person with dementia from their home to a nursing home

A DEDICATED webpage (www.dedicatedwerkwijze.nl) was developed that guides users to the appropriate support tools, sorted according to the themes and the place of care (home, nursing home or in transition). DEDICATED ambassadors explore learning goals within their teams, which then function as a basis to navigate the webpage and find the support they need. Most of the tools are available in a digital format that users can download and open on their digital device or print. In addition, we made videos for each of the tools that explains how to use them (in Dutch) (DEDICATED, 2021).

#### 4.1.2 DEDICATED training

The DEDICATED ambassadors play an important role in stimulating awareness about palliative care, introducing the DEDICATED approach to their colleagues and sustaining the application of the DEDICATED approach in practice. To prepare the ambassadors for the implementation of the DEDICATED approach within their care teams, we developed a training in co‐creation with professional educators. After completing the DEDICATED training, DEDICATED ambassadors:

1. are able to explain what palliative care means and why it is important for people with dementia,
2. are able to apply the DEDICATED approach in practice, identify learning goals within their teams and match the DEDICATED approach to these learning goals within their organizational contexts,
3. Have the knowledge and skills to enthuse and motivate their colleagues to apply the DEDICATED approach,
4. Participate in a peer‐to‐peer support group of ambassadors and learn how to implement, disseminate, critically reflect and sustain the DEDICATED approach together.

Professional educators provided ambassadors' training to a ‘first wave’ of DEDICATED ambassadors (i.e. HCPs from the development group). After completing the training, two ambassadors were asked to become DEDICATED trainers. The most important requirements for becoming a DEDICATED trainer were motivation and didactical experience to regularly train and present the DEDICATED approach to new ambassadors.

**FIGURE 4:**
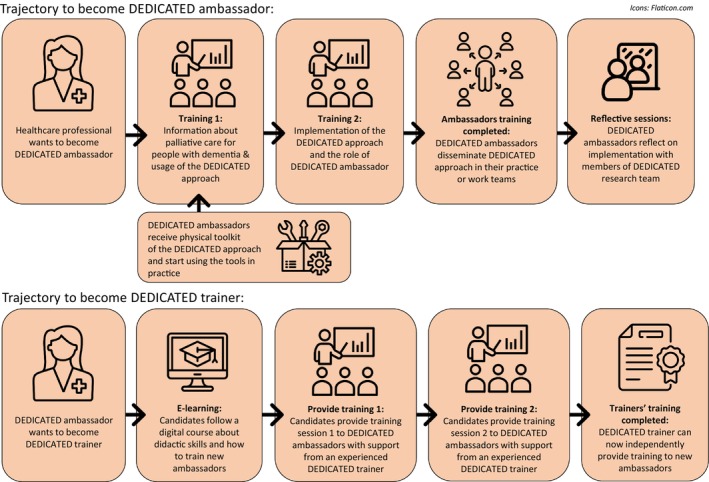
Delivery method of the DEDICATED approach to DEDICATED ambassadors and trainers.

DEDICATED ambassadors from the first wave helped to recruit a ‘second wave’ of ambassadors from the partner organizations. This group received the DEDICATED training from the DEDICATED trainers instead of professional educators. Figure 4 visualizes the training steps to become a DEDICATED ambassador and a DEDICATED trainer.

### 4.2 Results of the interviews with DEDICATED ambassadors

We had a 70% response rate (17/24) for the interviews. Seven interviewees were first‐wave ambassadors and ten were second‐wave ambassadors. Their mean age was 39.5 years (SD = 12.6, range 23–59), and 76% were nurses. On average, the first‐wave ambassadors had 8.9 years of experience in their current palliative care profession, and the second‐wave ambassadors had 3.4. Seven DEDICATED ambassadors did not participate in the interviews due to illness or lack of time. Table 2 shows the participants' characteristics.

**TABLE 2:** Characteristics of the interviewed DEDICATED ambassadors.

Participant	Age (in years)	Profession	Experience (in years)	Start ambassadorship
1	52	Consulting palliative care nurse	4	2019^b^
2	31	Quality nurse practitioner^a^	2	2019^b^
3	46	Nurse	23	2019^b^
4	47	Quality nurse practitioner^a^	4	2019^b^
5	57	Dementia case manager	3	2019^b^
6	36	Psychologist	13	2019^b^
7	59	Quality nurse practitioner^a^	13	2019^b^
8	59	Quality nurse practitioner^a^	4	2021^c^
9	23	Nurse	1	2021^c^
10	27	Dementia case manager	2.5	2021^c^
11	23	Nurse	2	2021^c^
12	43	Home care nurse	12	2021^c^
13	30	Home care nurse	4.5	2021^c^
14	33	Quality nurse practitioner^a^	2.5	2021^c^
15	28	Team manager care	2	2021^c^
16	30	Consulting palliative care nurse	2	2021^c^
17	49	Nurse	1	2021^c^

^a^ Nurses who provide care for people with dementia combined with a (quality) managing role.

^b^ First wave of DEDICATED ambassadors (i.e. development team).

^c^ Second wave of DEDICATED ambassadors.

We formulated the following five themes by analysing the interview transcripts:

- working with the tools as part of the DEDICATED approach,
- the training as part of the DEDICATED approach,
- the role of DEDICATED ambassadors,
- ambassadors' strategies to implement the DEDICATED approach,
- securing the DEDICATED approach for long‐term use.

Data S1 depicts the coding overview of the five main themes.

#### 4.2.1 Experiences of working with the tools as part of the DEDICATED approach

DEDICATED ambassadors experienced increased awareness about the goals of palliative care and when such can be conducted. Furthermore, the DEDICATED ambassadors shared that they became more conscious of the importance of ACP and that every person with dementia has a personal life story. Using the DEDICATED tools helped the ambassadors promptly explore the life story of a person with dementia and use this information in their care provision. For instance, by using photo cards and proactively starting a conversation about a person's needs or background, ambassadors knew when and how a person wanted to receive care. Ambassadors matched the care routine with these needs, eventually saving time and improving the person's quality of life.

Regarding the use of a person's life story, I notice that many colleagues now bring that up when someone is in distress. It often helps to talk about the past with the person. (P2, quality nurse practitioner)

I started to see the resident as a resident. As a person. A person with a life instead of a resident with a disease. (P1, consulting palliative care nurse)

The tools of the DEDICATED approach were considered pleasant to work with by the DEDICATED ambassadors. The aesthetics, practicality, and tangibility of the tools made them easy to use for a variety of care professionals in different situations. Several tools provided an opening to start conversations with persons with dementia, their loved ones and team members, such as the photo cards and dice. Other tools, such as the case discussion format, provided a structure for HCPs to discuss a specific situation or case. In addition, the description of the tools could be easily found on the website, which helped ambassadors when things were unclear. Some DEDICATED ambassadors also explained that they mainly used the more visual and tangible tools of the DEDICATED approach. This was generally because they found these tools more appealing, and they did not have the time to read large amounts of text.

I notice that there is more depth when discussing clients' cases. There is more structure, which helps obtain a broader impression of a resident. Previously, our structure was that all colleagues would randomly provide information for a case. Now, I notice that by applying the structure of the DEDICATED approach, specific aspects are discussed that normally would not be mentioned. (P16, consulting palliative care nurse)

The tools are very nice and clear. I think the cards with pictures look really good. They provide many opportunities because, as we know, language and wording becomes more difficult over time for people with dementia. Those pictures are really helpful for building connections and make it possible to start a conversation. (P15, team manager)

While the experiences with using the tools were mainly positive, some DEDICATED ambassadors indicated a few remaining hurdles. Even though the tools provided an opening for conversations about the end of life, the ambassadors still experienced uncertainty in finding the right timing to bring up this topic. In addition, talking about the end of life was perceived to be a sensitive topic, and it was difficult for ambassadors to assess if people with dementia and their caregivers were ready to have the conversation.

When you look at the numbers, you can see how dementia progresses and you know the average lifespan after the diagnosis. For people with dementia, this is mostly unclear and the question is how are you going to discuss this as a caregiver? I think that is really difficult. (P7, Dementia case manager)

#### 4.2.2 Experiences with the training as part of the DEDICATED approach

The DEDICATED ambassadors reported that the training sessions as very interactive and helped them to learn how to motivate colleagues, implement the tools of the DEDICATED approach and use the tools during the provision of palliative care. In addition, the training helped them reflect on their current ways of providing palliative care. This process created awareness about how to improve as a palliative care professional. The DEDICATED ambassadors were positive about the interactive elements of the training, such as discussing situations that occurred while providing palliative care for people with dementia.

I liked it. I liked that different organizations came together so that we could hear about others' approaches. That gave me ideas. It was quite interactive, which I also really liked. (P16, consulting palliative care nurse)

Due to COVID‐19, some of the DEDICATED training sessions were online, which interfered with the interactive approach to the training sessions. The DEDICATED ambassadors preferred having live training sessions.

I would be nicer if you could sit together. That really brings about more interaction. I really missed that the face‐to‐face interaction and being together. (P4, quality nurse practitioner)

I understand that in the future we will often discuss things online, but I think that palliative care is a topic that should be discussed in person. (P6, psychologist)

While the experiences with the DEDICATED training were mainly positive, the DEDICATED ambassadors indicated that they needed more training on strategies to adequately implement and disseminate the DEDICATED approach in their own care organization. Furthermore, some DEDICATED ambassadors felt that the training sessions were too long.

I think… about the implementation, I already spoke about that… that it would be nice if there was more information supporting us in the implementation. (P11, nurse)

#### 4.2.3 Experiences with the role as a DEDICATED ambassador

The DEDICATED ambassadors indicated that they felt responsible for proactively implementing and stimulating the usage of the DEDICATED approach within their care organizations. DEDICATED ambassadors explained that they need support from their managers. For example, one DEDICATED ambassador mentioned that the quality management department was proactively involved in making the DEDICATED approach known within the organization.

We observed a difference between the first‐wave and second‐wave ambassadors in terms of experienced ownership of the DEDICATED approach. DEDICATED ambassadors from the first wave (i.e. the HCPs that co‐created the DEDICATED approach) had a clearer view of what being a DEDICATED ambassador entailed and felt more ownership over the tools and the implementation process. They undertook more in‐depth actions to make the DEDICATED approach known within their organization. For example, first‐wave consulting palliative care nurses and quality nurse practitioners used their knowledge about palliative care to inform colleagues on integrating the DEDICATED approach into daily palliative care activities.

As DEDICATED ambassador, you can help answer questions, but you can also immediately put the tools to use and position them in a care team as part of the knowledge about palliative care. (P4, quality nurse practitioner)

Two DEDICATED ambassadors (from the second wave) explained that they found it difficult to fulfil their role as DEDICATED ambassadors because they worked in the home care setting or as a dementia case manager. While nursing home care teams have regular meetings, home care professionals often do not meet each other during working days. This made it difficult for them to introduce the DEDICATED approach.

In the role of DEDICATED ambassador, it is difficult to transfer the approach to other colleagues, because a lot of the time we work alone. (P5, dementia case manager)

Many factors influenced the role of the DEDICATED ambassadors. First, DEDICATED ambassadors explained that changes in the working routine (such as the introduction of the DEDICATED approach) were not always easily accepted by HCPs because they deviated from existing methods. Second, staff shortages due to the COVID‐19 pandemic limited the amount of time that DEDICATED ambassadors and their colleagues had to implement the DEDICATED approach.

They want to do it, they are willing to work with it, and they are excited. It is not used yet because… yeah… it is still in their system that as a nurse you only have to wash, dress, or feed the patients. (P17, nurse)

Yeah, I don't know how to judge the teams at the moment. There has been a lot of restlessness and changes and… you name it. Not everything is easily accepted. New methods are quite difficult to implement. (P8, quality nurse practitioner)

#### 4.2.4 Ambassadors' strategies for implementing the DEDICATED approach

The DEDICATED ambassadors used various strategies to present and implement the DEDICATED approach into their healthcare organizations. For instance, ambassadors facilitated interactive meetings to let team members experiment with the DEDICATED approach and referred them to the website to obtain the associated information and examples of the tools.

Well, I first introduced it during work meetings. We have had work meetings during the pandemic, but in small groups of four or five persons. So for four days in a row, I explained what DEDICATED entailed and what I am doing. (P13, home care nurse)

Furthermore, the DEDICATED ambassadors actively involved colleagues and the management of the healthcare organization in the process of implementing the DEDICATED approach. Colleagues who were eager and motivated to work with the DEDICATED approach were given the task to disseminate the DEDICATED approach in different units or organizations. By empowering and involving team members and giving them the task to motivate other colleagues, the DEDICATED approach became better known within care units or healthcare organizations.

If you are doing the implementation alone in a team, it does not go well. So you really need your colleagues to help with that. (P16, consulting palliative care nurse)

I knew people who were open to using it, so I took them with me in the process. Then, they told other colleagues about it, because that's who they are. And then, during a team meeting, I addressed the topic and said: “this is what I'm currently doing; if you have any questions, please come to me.” So, if they needed anything or needed more explanation, they knew how to find me. So it is word of mouth publicity. (P9, nurse)

#### 4.2.5 Securing the DEDICATED approach for long‐term use

Some DEDICATED ambassadors reported that tangible tools, such as photo cards or chatter jars, were more often used than digital tools or on‐paper guidelines. To maintain the usage of the DEDICATED approach, ambassadors frequently mentioned that they want to keep attending regular face‐to‐face meetings with other DEDICATED ambassadors from different organizations to reflect on the provision of palliative care and the usage of the DEDICATED tools. Ambassadors suggested that a meeting platform could be created for ambassadors to contact each other and conduct reflective meetings. This approach could help them to sustain using the DEDICATED approach and gain more knowledge from other organizations and professionals.

Yes, regular meetings prevent it from fading away, right… Then it sticks in your memory like: “oh, we have a meeting planned.” Those meetings do not have to be every month, but I think that once every six months would be good. And yeah… I would really like that, and I also think that we can really learn from it. (P15, team manager)

The DEDICATED ambassadors needed to pay close attention to the linkage of the DEDICATED approach to their organization's policy to sustain the DEDICATED approach for long‐term use.

And perhaps for me, a point of attention is that I need to constantly refer back to the DEDICATED approach. (P4, quality nurse practitioner)

## 5 DISCUSSION

This study comprised two parts. The first entailed explicating the co‐creation of the DEDICATED approach. The second entailed exploring the experiences of the DEDICATED ambassadors in co‐creating and implementing the DEDICATED approach. Overall, the ambassadors described that co‐creating and implementing the DEDICATED approach was a positive experience that stimulated their self‐development as professional. In addition, ambassadors who co‐created the DEDICATED approach particularly expressed feelings of ownership. The ambassadors shared that the DEDICATED approach helped to create more awareness about when and how to provide palliative care for people with dementia. Throughout the interviews, ambassadors expressed specific needs for sustaining the utilization of the DEDICATED approach. They emphasized the importance of frequent meetings to exchange experiences and learn from fellow DEDICATED ambassadors. Additionally, they indicated a need for increased support from their managers to effectively implement the DEDICATED approach. Lastly, they underscored the necessity for additional training, particularly in the context of home care settings.

Prior to developing the DEDICATED approach, we investigated palliative care needs from the perspective of people with dementia, their loved ones and HCPs. From the results of this elaborate needs assessment, we identified six overall key themes that are the foundation of the DEDICATED approach. A co‐creative design was used to investigate these palliative care needs, derive the key themes from the needs assessment and build the DEDICATED approach. Co‐creative working methods (i.e. involving the end‐users in research and co‐creation) have been found to encourage feelings of ownership and motivation to implement and sustain the intervention (Langley et al., 2018). This observation is similar to our findings: all the interviewed DEDICATED ambassadors felt ownership in the implementation of the approach and involved team members in it. However, the results of the interviews also indicated several differences in terms of the extent of ownership felt by the DEDICATED ambassadors in implementing and disseminating the DEDICATED approach: DEDICATED ambassadors from the first wave (that co‐created the DEDICATED approach) took more elaborate actions to make the DEDICATED approach familiar within their work environment. For example, they implemented the DEDICATED approach in multiple wards or directly involved policymakers and management to integrate the DEDICATED approach into the organization's current ways of working. The ambassadors of the second wave (that were not involved in the co‐creation) reported more challenges in implementing the DEDICATED approach in their organizations. For example, implementation of the DEDICATED approach was mostly conducted during the high tide of the COVID‐19 pandemic and the priorities of nurses was often not aimed at introducing new working methods within their organization or team since they were dealing with a crisis situation (Rutten et al., 2022). After the COVID‐19 period, implementation challenges remained due to overburdened staff, sick leave and job changes (Rutten et al., 2022). These aspects could influence the implementation of the DEDICATED approach. In addition, some ambassadors experienced resistance from co‐workers for using new working methods, such as the DEDICATED approach. The ambassadors highlighted that the attitude of team members towards using the DEDICATED approach was seen as an important aspect for its successful implementation. Literature regarding the implementation of new interventions confirms that the attitude of colleagues and their motivation to adapt to a new way of working is one of the most important factors for successful implementation (Colditz et al., 2012; Grol et al., 2005). To prevent reluctance and resistance from team members regarding the DEDICATED approach, we asked the DEDICATED ambassadors about their implementation strategies. They mostly involved enthusiastic team members as a ‘buddy’ in the implementation process. Ambassadors highlighted that this helped create more support for using the DEDICATED approach and gave them more confidence during the introduction. This provided the DEDICATED ambassador with more confidence to introduce the approach. Some ambassadors also involved managers to embed the DEDICATED approach into daily practice and the organization. A scoping review by Collingridge Moore et al. (2020) confirms that strategies such as: (1) raising awareness about palliative care, (2) embedding new interventions in the current way of working, (3) involving managers that can help to facilitate implementation and (4) asking team members to help in the implementation are effective ways to implement new palliative care interventions. Other aspects that could influence the implementation by the second‐wave ambassadors are that they were not involved in the co‐creation process and therefore needed more time to become familiarized with the application of the DEDICATED approach before implementing it within their care organizations. Another explanation for the implementation challenges could be that, on average, the DEDICATED ambassadors from the first wave had more years of working experience in their current profession than the second wave of ambassadors. Whitehead et al. (2022) found that the extent of working experience could influence the implementation of new interventions in practice. Successful implementation depends on adequate communication between team members (e.g. more insight into team dynamics and working with people with dementia in a palliative phase) (Grol et al., 2005; Fernandez et al., 2019). Building this expertise and communicational skills takes time and depends on the working experience HCPs have (Gillett et al., 2016; Norouzinia et al., 2016). Therefore, the shorter work experience could have contributed to the challenges of the second‐wave ambassadors in implementing the DEDICATED approach. A third reason could be that the COVID‐19 pandemic influenced the implementation in the second wave.

Other challenges regarding the implementation lie within the different settings in which ambassadors implemented the DEDICATED approach. For example, our results indicate that DEDICATED ambassadors who worked in the extramural setting (e.g. as home care nurses or as dementia case managers) experienced more challenges when implementing the DEDICATED approach in their care teams because they work individually and often do not physically meet up with team members during work days, resulting in less communication within these home care teams than in care teams that provide care within the nursing home setting. The limited time of extramural care professionals to physically meet and collaborate with team members could negatively influence their motivation to use new working methods (Noguchi‐Watanabe et al., 2021). Physical meetings were considered more effective to introduce the DEDICATED approach because team members could experiment with the tools and ask questions if necessary. The COVID‐19 pandemic also hampered the ability of DEDICATED ambassadors (especially those from the second wave) to conduct physical meetings to introduce the DEDICATED approach.

DEDICATED ambassadors also shared their insights regarding the value added by the DEDICATED approach. The training component of DEDICATED raised awareness about palliative dementia care, including the timing and methods of its provision, centralizing the wishes, needs and values of individuals with dementia, and initiating end‐of‐life conversations. By creating awareness about what palliative care entails, HCPs felt more competent and had improved literacy regarding palliative care for people with dementia. Li et al. (2020) highlight that HCPs may lack knowledge or literacy regarding palliative care for people with dementia, making it important that newly‐developed interventions, such as the DEDICATED approach, address what palliative care entails. Meier et al. (2017), describe that facilitating interactive training sessions and peer discussions can contribute to the knowledge transfer about palliative care between HCPs. Studies have also emphasized the value of peer learning and team interactions, such as the buddy system, over traditional teaching methods (Parkin, 2006; Stone et al., 2013). Additionally, DEDICATED ambassadors found the DEDICATED tools to be accessible and user‐friendly, with a preference for visual and tangible resources over more textual tools. Previous research has suggested that tangible and visually appealing tools aid in better comprehension of the information presented (Chang & Bourgeois, 2020; Lipkus, 2007; Samuelsson & Ekström, 2019). Accessible tools can positively influence HCPs' thinking and discussions on topics like the end‐of‐life wishes and needs of individuals with dementia, as well as support them in conducting ACP discussions and making shared decisions (Lipkus, 2007; Roodbeen et al., 2020).

### 5.1 Limitations

To identify the experiences with developing and implementing the DEDICATED approach, we specifically chose to interview the DEDICATED ambassadors, as they were closely involved in the co‐creation and implementation processes. While this may be considered as a strength of this study, it could also be a limitation. Because DEDICATED ambassadors are motivated and enthusiastic to be involved in the co‐creation and implementation, they could have given socially desirable answers during the interviews, possibly biasing our results. However, our participant group represented a mix of first‐wave ambassadors (co‐creators) and second‐wave participants (less involved in co‐creation), spanning various healthcare disciplines and settings, enhancing the relevance of the results for diverse professionals and contexts. Nevertheless, other end‐users of the DEDICATED approach (e.g. HCPs not serving as ambassadors) may have had different perspectives. Additionally, the study did not capture the perspectives of people with dementia and family caregivers regarding the impact of the DEDICATED approach on palliative care, potentially yielding different results. Another limitation was that the researchers who conducted the interviews were involved in developing and disseminating the DEDICATED approach. This may have influenced the interviewing and data interpretation. However, two researchers (*one* of which was not involved in the DEDICATED project) individually analysed and coded the transcripts (Jonsen & Jehn, 2009).

## 6 CONCLUSION

This study outlined the development of the DEDICATED approach and assessed its implementation experiences. Important lessons learned from this research include (1) involving HCPs extensively in co‐creation fostered ownership, positively impacting approach implementation, (2) implementation within different settings requires context‐specific strategies and may be positively influenced when DEDICATED ambassadors receive support from team members and managers, (3) the training and tools of the DEDICATED approach were experienced as helpful by the ambassadors in providing palliative dementia care and (4) regular meetings among DEDICATED ambassadors can be beneficial to sustain usage of the DEDICATED approach. Other implementation studies may also benefit from these findings. For example, by applying the strategies of the DEDICATED ambassadors to foster the implementation of new interventions. As palliative care gains importance, collaborative efforts, such as the DEDICATED approach, are helpful for improving the quality of life for those with life‐limiting conditions and improving palliative care provided by HCPs.

## AUTHOR CONTRIBUTIONS

Made substantial contributions to conception and design or acquisition of data or analysis and interpretation of data: Jesper M. A. Biesmans, Sascha R. Bolt, Judith M. M. Meijers. Involved in drafting the manuscript or revising it critically for important intellectual content: Jesper M. A. Biesmans, Sascha R. Bolt, Daisy J. A. Janssen, Toon Wintjens, Chandni Khemai, Jos M. G. A. Schols, Jenny T. van der Steen, S.M.G., Judith M. M. Meijers. Given final approval of the version to be published. Each author should have participated sufficiently in the work to take public responsibility for appropriate portions of the content: Jesper M.A. Biesmans, Sascha R. Bolt, Daisy J. A. Janssen, Toon Wintjens, Chandni Khemai, Jos M. G. A. Schols, Jenny T. Van Der Steen, Sandra M. G. Zwakhalen, Judith M. M. Meijers. Agreed to be accountable for all aspects of the work in ensuring that questions related to the accuracy or integrity of any part of the work are appropriately investigated and resolved: Jesper M. A. Biesmans, Sascha R. Bolt, Daisy J. A. Janssen, Toon Wintjens, Chandni Khemai, Jos M. G. A. Schols, Jenny T. Van Der Steen, Sandra M. G. Zwakhalen, Judith M. M. Meijers.

## FUNDING INFORMATION

This research was funded by The Netherlands Organization for Health Research and Development (ZonMw) (project no. 10200012110004). ZonMw had no role or influence in the conducted research or its findings.

## CONFLICT OF INTEREST STATEMENT

The authors confirm that they have no affiliations or involvement in any organization or entity with any financial interest or non‐financial interest in the subject matter or materials discussed in this manuscript.

## PEER REVIEW

The peer review history for this article is available at https://www.webofscience.com/api/gateway/wos/peer‐review/10.1111/jan.16285.

## Supporting information


Data S1.

Data S2.

## Data Availability

The data that support the findings of this study are available from the corresponding author upon reasonable request.
