# Supplementary material for: Desired dementia care towards end of life: Development and experiences of implementing a new approach to improve person‐centred dementia care
Source: J Adv Nurs. 2024 Jun 24;81(11):7152–66. doi: 10.1111/jan.16285 (PMC12535342; doi:10.1111/jan.16285)
Supplement: Supplementary file 1 — Data S1. [file JAN-81-7152-s002.docx]

# Supplement 1: Topic list interviews

**Ask characteristics:**

- Male/female
- At which organization and which department/ward or unit do you work?
- What is your current profession/function within the organization?
- How many years of experience do you have in this function?
- Since when did you become DEDICATED ambassador?

**Content-related questions:**

1. How did you experience working with the DEDICATED approach?
   1. Wat do you think about the materials/tools?
   2. Wat do you think about the training?
   3. Which aspects contributed to the application of the DEDICATED approach in practice?
   4. Which aspects obstructed the application of the DEDICATED approach in practice?
   5. How did COVID-19 influence the application of the DEDICATED approach?
   6. How did you deal with these obstructing factors? (including COVID-19)
2. How did you communicate and share the DEDICATED approach to your colleagues?
   1. Which steps did you take in this process?
   2. Did you manage to reach your complete team of colleagues? If yes, how did you manage this? If no, what could have caused this?
   3. Which aspects were contributing to the sharing and communicating about the DEDICATED approach?
   4. Which aspects were obstructing to the sharing and communicating about the DEDICATED approach?
3. Which effects did you see from working with the DEDICATED approach?
   1. How did the DEDICATED approach influence you as healthcare professional? Can you give an example?
   2. How did the DEDICATED approach influence the collaboration with your colleagues? (and with colleagues from other disciplines?) Can you give an example?
   3. How did the DEDICATED approach have an influence on people with dementia and their relatives/caregivers? Can you give an example?
   4. *If there were little effects (on one or more of the before named aspects): What could be the cause for this in your opinion?*
   5. What is, in your opinion, the adding value of the DEDICATED approach for healthcare professionals, people with dementia and relatives?
4. Looking back at your role as DEDICATED-ambassador…
   1. How did you experience this role?
   2. How did you prepare yourself to work and use the DEDICATED approach?
   3. Did you grow in your ambassadorship? What was contributing to this?
   4. To what extend did you feel supported by your employer/organization to fulfill this role? (For example, were you facilitated and encouraged by management teams?)
   5. How are you going to continue as an ambassador? What or whom do you need to continue?
   6. Do you want to keep being involved? If yes, how?
   7. Do you have tips for future DEDICATED ambassadors?
5. Which aspects should we take into consideration for the future of DEDICATED?
   1. Which materials do you think should be altered, adjusted or improved? Which adjustments?
   2. How can the training be improved?
   3. Do you have other recommendations or comments that you would like to mention?
6. Are there other aspects that are not yet mentioned and you would like to discuss?
