# Supplementary material for: Desired dementia care towards end of life: Development and experiences of implementing a new approach to improve person‐centred dementia care
Source: J Adv Nurs. 2024 Jun 24;81(11):7152–66. doi: 10.1111/jan.16285 (PMC12535342; doi:10.1111/jan.16285)
Supplement: Supplementary file 2 — Data S2. [file JAN-81-7152-s001.docx]

# Supplement 2: Coding overview

| Experiences with the training as part of the DEDICATED approach. | Experiences with the role as a DEDICATED ambassador. |
| --- | --- |
| - DEDICATED-training creates awareness about palliative care - DEDICATED-training connects organizations and professionals - DEDICATED-training takes a lot of time - Positive about learning from other organizations | - Actively disseminating en promoting the DEDICATED-approach - DEDICATED-ambassadors actively use the materials - Dementia case managers work individually - Meeting other ambassadors - Positive about communication between research group and end users - Unclear what the role as DEDICATED-ambassador entails |
| Ambassadors’ strategies to implement the DEDICATED approach. | **Experiences of working with the materials as part of the DEDICATED approach.** |
| - Being supported/facilitated by the employer and care organization - COVID-19 hinders implementation - Create awareness about the usage of the DEDICATED-approach - Creating support for using the DEDICATED-approach - Difficult to convince colleagues - Embed the DEDICATED-approach in the care process instead of - Enthuse and motivate colleagues to use the DEDICATED-approach. - Facilitate a clinical lesson about the DEDICATED-approach   focussing on individual care professionals   - High work pressure and time shortage - Highlight the current way of working during the introduction of the DEDICATED-approach - Introduce the DEDICATED-approach using an internal platform for employees - Introduce the DEDICATED-approach with the use of a poster - Introducing the DEDICATED-approach during team meetings - Involve colleagues in the implementation - Involve managers/Board of Directors in the implementation - Involve quality management in the implementation - Learning to use the DEDICATED-approach by experimenting in different situations - Make colleagues curious by showing and using the DEDICATED-approach - Make colleagues curious by showing the website - Personnel shortage and changes - Proactively implement the materials - Reach out to colleagues by e-mail - Show colleagues the benefit of the DEDICATED-approach - Some colleagues are unwilling or unenthusiastic - Start the implementation small | - Awareness about own roles a healthcare professional - Awareness about person-centered care - Awareness about palliative phase of the patient - Awareness about the needs of a care team - Awareness about using the needs and wishes of a patient to provide palliative care - Chatter jar difficult to use - DEDICATED-approach is accessible and widely usable - DEDICATED-approach stimulates conversations between residents - Dices with photos are too small - End-of-life care is a difficult topic - Involve dementia case managers in the palliative care provision - Involving relatives in the palliative care provision - Material helps to identify responsive behaviour - More in-depth discussion about patients - No effect in the interprofessional collaboration - Paper materials are disappearing to the background - Photo cards difficult to use - Photo cards make it easier to start conversations - Positive about tangibility of materials - Positive about the guidelines for interprofessional collaboration - Seeing the patient as a person - Supportive in current way of working - The DEDICATED-approach is different for every patient - Using background stories can be timesaving |
| Securing the DEDICATED approach for long-term use. |  |
| - In need of physical meetings - In need of regular intervision meetings - Evaluate DEDICATED with multiple team members - Evaluate the usability of materials - Planned meetings with researchers or ambassadors improve continuity and prevents the DEDICATED-approach from moving to the background - Proactively securing the usage of the DEDICATED-approach |  |
